# Should Mumps Be Higher Up on the Public Health Agenda in India? A Concern for Global Health Security

**DOI:** 10.3390/medsci6030062

**Published:** 2018-08-07

**Authors:** Syed Manzoor Kadri, Saleem-ur Rehman, Kausar Rehana, Ailbhe Helen Brady, Vijay Kumar Chattu

**Affiliations:** 1 Directorate of Health Services, Kashmir 190001, India; kadrism@gmail.com (S.M.K.); padhsk@gmail.com (S.-u.R.); rkousar06@gmail.com (K.R.); 2 Global Health Research, 98 Temple Street, London E26QQ, UK; bradyailbhe@gmail.com; 3 Department of Paraclinical Sciences, Faculty of Medical Sciences, The University of the West Indies, St. Augustine, Trinidad and Tobago

**Keywords:** mumps, universal immunization programme, vaccination, disease surveillance, global health security, paramyxovirus

## Abstract

Mumps is a public health problem on a global scale caused by mumps virus, a member of family paramyxoviridae. An effective form of vaccination exists and is incorporated into routine immunization schedules in over 100 countries, usually in the form of the Measles, Mumps and Rubella (MMR) vaccine. This is not the case in India, as mumps is not viewed as a significant enough public health problem by the government to warrant such an intervention. This original research paper discusses about outbreaks of mumps in Kashmir, India and aims to add to the body of literature to support the routine immunization with the mumps vaccine. From July to September 2017, there were 15 outbreaks and 260 cases of mumps recorded in the region by the Integrated Disease Surveillance Programme (IDSP). We conclude that the Indian Government should include the MMR vaccination in the Universal Immunization Programme. This would result in clinical and economic benefits by reducing outbreaks and associated morbidity of mumps, in addition to tackling the recognized morbidity and mortality of rubella and measles. To support the global health security, there is a great need to strengthen surveillance, adhere to the World Health Organization’s International Health Regulations (IHRs), and pay attention to emerging and re-emerging infectious agents, including paramyxovirus group.

## 1. Introduction

The Universal Immunization Programme (UIP) of India does not include the measles, mumps and rubella (MMR) vaccination. It currently vaccinates against measles, however not rubella or mumps [1]. By presenting data collected from the Kashmir region of India, this paper aims to add to the body of literature supporting inclusion of the mumps vaccination in the UIP on the basis that it is a significant public health problem. We posit that it would be worthwhile ensuring routine MMR vaccination, thereby also ensuring robust immunization against measles and rubella, and tackling associated morbidity, mortality, and economic sequelae.

Mumps is a viral infection caused by a paramyxovirus. The virus spreads through direct contact with respiratory secretions, saliva, or through fomites. It replicates in the upper respiratory tract and the average incubation period is 16 to 18 days. Mumps primarily affects the salivary glands, causing pain, tenderness, and swelling in one or both parotid glands. Prodromal symptoms may precede parotitis by several days, consisting of low grade fever, myalgia, anorexia, malaise, and headache. The parotitis typically lasts for at least two days and further spread is enhanced by longer and closer contact with an infected individual. Therefore, it is advised that an infected individual should avoid contact with others from the time of diagnosis until five days post-onset of parotitis [2]. Vaccination is recognized as the best way to prevent mumps. The first vaccine against mumps was licensed in the United States in 1967—it is a live attenuated vaccine, given as a simple subcutaneous dose, usually in form of the MMR vaccine. According to the US Centers for Disease Control, two doses of the mumps vaccine are 88% effective at preventing the disease, and one dose is 78% effective. Vaccinated individuals are less likely to present severe symptoms or complications than under vaccinated or unvaccinated individuals [3]. The World Health Organization (WHO) and Indian Association of Paediatrics (IAP) recognize the vaccine as a highly effective way of preventing infection—the IAP includes it in its vaccination schedule [4].

## 2. Methodology

### 2.1. Disease Surveillance in India

The Integrated Disease Surveillance Programme (IDSP) of India aims to strengthen and maintain decentralized laboratory-based disease surveillance systems for epidemic-prone diseases to monitor disease trends and to detect and respond to outbreaks promptly through trained Rapid Response Teams (RRTs) [5]. The Kashmir IDSP Division collected data for mumps outbreaks in Kashmir from January 2017 to September 2017, and the resulting study is presented in this paper.

### 2.2. Universal Immunization Programme of India

The Universal Immunization Programme began in India in 1985. It was an extension to the Expanded Programme for Immunization (EPI), which endeavoured to provide recommended vaccines against tuberculosis, polio, and other diseases for all Indian children. The national policy of immunization of all children during the first year of life with DPT (Diphtheria, Pertussis and Tetanus), OPV (Oral Polio Vaccine) and BCG (Bacillus Calmette-Guerin)—with the series of primary vaccinations to be completed prior to the age of one—was adopted in 1978 with the launching of the EPI to increase the immunization coverage in infancy to 80%. The Ministry of Health and Family Welfare was responsible for the programme, with significant support from the international community.

The UIP is one of the largest in the world in terms of quantities of vaccine used, the number of beneficiaries, the number of immunization sessions organized, the geographical spread, and the diversity of areas covered [1]. Advances in immunization coverage have occurred, however not without management challenges. The Programme has not met the target of coverage for all children [6].

The current vaccination schedule under the UIP is shown in Table 1 below.

At present, the vaccination schedule (Table 1) does not include mumps [6].

The reasoning behind this is that it is not a significant enough public health problem. Despite being a widely prevalent disease in the Country, it is considered as an insignificant public health problem mainly due to poor documentation of clinical cases and a lack of published studies. This paper endeavours to add to the body of literature which supports introduction of mumps vaccine as part of the UIP vaccination schedule [7].

### 2.3. Arguments against Vaccination

Globally, we see the issue of waning immunity, for instance an outbreak at a Kansas University in 2006 where there was a two-dose MMR policy [8]. In 2009 and 2010, Orthodox Jewish communities in the United Kingdom experienced outbreaks. Investigation found that intense exposures—especially amongst boys in schools—facilitated transmission and overcame vaccine-induced protection in these patients [9]. A study in France found that mumps immunity waned with increased time since vaccination. The odds of mumps infection increased for those with two-dose MMR by 10% per year passed since the time of receiving the second dose [10]. Additionally, there is the consideration that other diseases, for instance measles, require financial resources more than mumps, due to the greater associated morbidity, mortality and economic sequelae.

### 2.4. Integrated Disease Surveillance Programme and Mumps

The IDSP has been recording outbreaks of diseases including mumps. The epidemiological methods employed in this instance involved collecting the number of outbreaks and diagnosing cases based on clinical signs and symptoms. The WHO-recommended clinical case definition is: ‘acute onset of unilateral or bilateral tender, self-limited swelling of the parotid or other salivary gland, lasting two or more days and without other apparent cause’ [11].

Door-to-door visits occurred for patients in the affected villages and schools, examining for signs and symptoms. Additionally, the living conditions and surroundings of the inhabitants were examined. The current rate of cases was compared to the background rate, and the outbreak described with respect to time place and person.

From July 2017 to September 2017, 15 outbreaks were recorded in the Kashmir region by the IDSP, totalling 260 cases (Table 2) as shown below. The outbreaks were across seven districts in 15 villages. The minimum number of cases in an outbreak was seven and the maximum 50, averaging 17 patients per outbreak.

**Figure 1:**
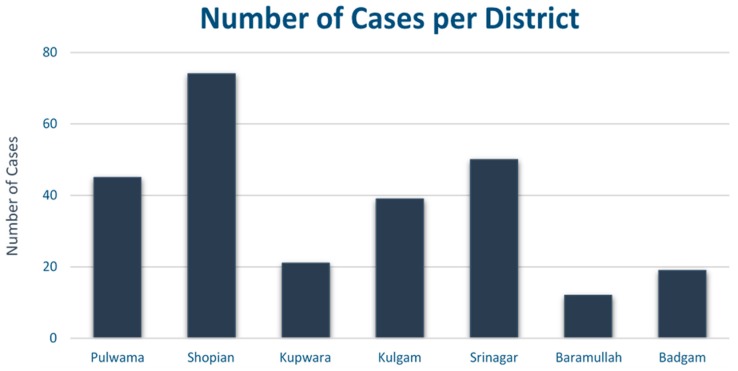
Bar chart showing total number of cases per district.

The largest outbreak was in an educational institution, in keeping with findings from existing literature which indicates that group settings associated with educational institutions are at increased risk of outbreaks, be they small or large [12]. The majority of outbreaks were recorded in the Shopian District, totalling five outbreaks with 74 cases overall (Figure 1).

### 2.5. Mumps Outbreak and Indepth Analysis of Shopian District

To provide more context, an outbreak in the Shopian District that was detected on 15 August 2017 will be looked at in detail. Twenty cases were identified in the Bal-Ashram Institute, which has a total enrolment of 55 people. The first case was reported on 14 August 2017 and clustering of cases occurred on 15 August 2017. The current rate of infection—36.36% (20/55)—was compared to background rate—0%—by reviewing weekly IDSP data for the years 2016 and 2017.

Interventions made due to the outbreak took the form of an infographic and door to door surveys relaying education about transmission and prevention of the disease. Advice comprised:

- Keep affected children isolated from gathering.
- Wash hands thoroughly with soap.
- Keep affected children at home for five days and avoid close contact with others in house.

**Figure 2:**
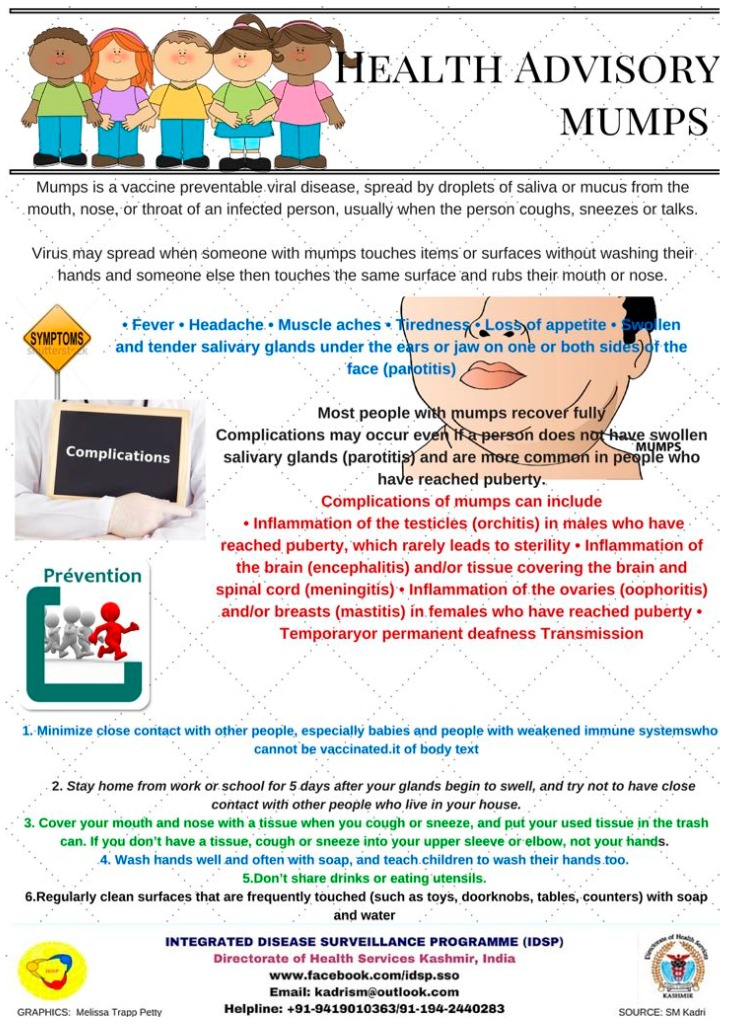
Integrated Disease Surveillance Programme mumps health advisory.

**Figure 3:**
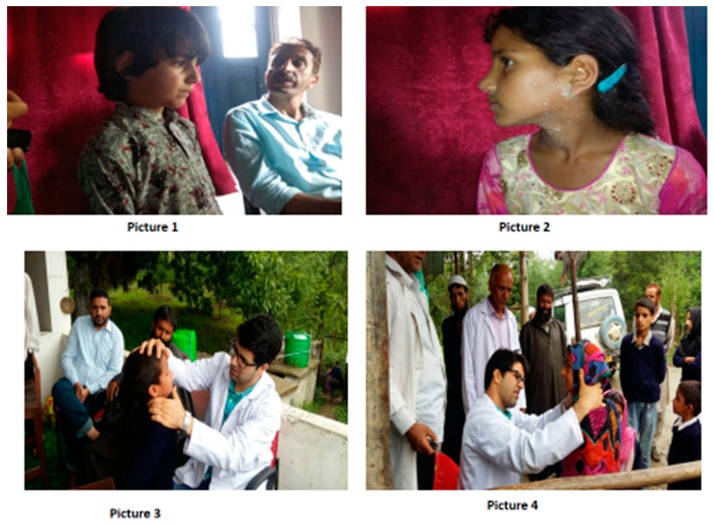
Clinical examination and follow-up checkup for affected children in the Shopian district.

Additionally, health advisory pamphlets were distributed and pasted to public place walls (Figure 2), a team of doctors was deployed to the area to identify and treat patients, schools were advised to avoid overcrowding, and field staff were instructed to follow up affected children regularly until they were fully recovered (also see Figure 3).

This outbreak only affected children, resulted in no hospitalizations or deaths, and no children developed major long-term sequelae.

## 3. Discussion

Mumps vaccination is a proven way of preventing infection with the virus, reducing severity of disease if contracted, and reducing transmission. Two doses of the vaccine are 88% effective at protecting against mumps and one dose is 78% effective [3]. Waning immunity is a recognized issue, however on balance with the benefits of vaccination, this paper argues that this is not reason enough to choose not to routinely vaccinate.

During mumps outbreaks in highly vaccinated communities, the proportion of cases that occur amongst the vaccinated population may be high. However this should not result in defaulting to the belief that the vaccine is not effective. The effectiveness of the vaccine is assessed by comparing the rate of infection in the vaccinated cohort to the rate of infection in the non-vaccinated cohort. In outbreaks in highly vaccinated populations, the non-vaccinated cohort tends to have a greater infection rate than the vaccinated cohort [3].

Given the existence of the MMR vaccination, which is used in many countries globally, we suggest that the benefits of ensuring vaccination against mumps in addition to rubella and measles would be a plausible public health intervention that would have highly significant clinical and economic benefits. It would ensure vaccination against mumps in addition to greater immunization rates against measles and rubella.

### 3.1. Global Scenario

Studies have shown that two-dose MMR coverage reduces disease severity and transmission alongside a reduction in complications and hospitalization [9,13]. Vaccination against mumps has been mandatory in Poland since 2003, ensuring two-dose MMR coverage. Systematic execution of ensuring mumps vaccination resulted in a significant decrease in the number cases and further decline is expected [14]. By 2002 the mumps vaccination was included in the routine immunization schedule of 121 countries/territories. According to the WHO, although the case-fatality rate of mumps encephalitis is low and overall mortality is 1/10,000 cases, permanent sequelae occur in about 25% of encephalitis cases. Mumps is a leading cause of acquired sensorineural deafness among children, affecting approximately 5/100,000 mumps patients. Additionally, mumps infection during the first 12 weeks of pregnancy is associated with a 25% incidence of spontaneous abortion [11,15].

In 2017, the United States reported more than 6000 cases of contagious viral illness and there were 150 outbreaks that affected more than 9000 people across the country between January 2016 and June 2017. Nearly half of these outbreaks occurred in university settings where young adults were at the highest risk for mumps. A majority of 78% of patients had vaccination of which 70% had received two doses of the MMR vaccine. This rise in cases of mumps has prompted the US panel to endorse third vaccine dose [16].

In countries where vaccination was introduced and high coverage was sustained the incidence of the disease has dropped tremendously and circulation has stopped. In countries where vaccination was not introduced the incidence of mumps remains high, mostly affecting children aged 5–9 years [11,15]. Complications from mumps infection can include encephalitis, meningitis, painful swelling of the testicles or the ovaries, pancreatitis and hearing loss.

### 3.2. Issues of Global Health Security

The main aim of global health security is to make our interconnected world safe from infectious disease threats. The framework emphasizes for global preparedness and response to emerging infectious diseases. Control of the international spread of infectious diseases is the primary and historical responsibility of WHO [17]. The WHO’s IHRs, provide the legal instrument for doing so as these regulations are internationally-agreed set of rules for consistent, proactive, coordinated and multilateral collaborative efforts to address any potential global health security threat that may spread beyond borders [18]. Protecting the world from infectious disease threats requires that national governments share the responsibility of serving those most in need, wherever they live. The group of paramyxoviruses are also listed among the group of emerging and re-emerging infectious agents which have the potential to cause epidemics, and they need to be dealt with seriously by ensuring sustainable surveillance systems.

## 4. Conclusions

The Indian Government proposes that mumps is not a significant enough public health problem to warrant routine immunization, however the data presented from Kashmir suggests otherwise. Environmental methods alone are clearly not preventing outbreaks. Whilst the case study of the Shopian district did not result in any hospitalizations or deaths, there is still recognized morbidity associated with mumps infection and potential lifelong sequelae for infected individuals, which is a public health concern. Whilst this paper acknowledges that there are other communicable diseases with greater associated morbidity and mortality, it proposes that given the existence of the MMR vaccination, mumps is a virus that could easily be vaccinated against in combination with measles and rubella, as occurs in over 100 countries worldwide that routinely vaccinate against the virus. This paper should be taken into account alongside other literature within the body of evidence that proposes mumps as a significant enough public health problem to warrant investment in prevention through vaccination.

## Figures and Tables

**Table 1:** Universal Immunisation Programme (UIP) vaccination schedule.

Vaccination	Timing
Bacillus Calmette–Guerin	One dose at birth (up to one year if not given earlier)
Diphtheria, pertussis, tetanus toxoid	Five doses; three primary doses at 6, 10 and 14 weeks and two booster doses at 16–24 months and 5 years of age
Oral polio vaccine	Five doses; zero doses at birth, three primary doses at 6, 10 and 14 weeks and one booster at 16–24 months of age
Hepatitis B vaccine	Four doses; zero doses within 24 h of birth and three doses at 6, 10 and 14 weeks of age
Measles	Two doses; first dose at 9–12 months and second dose at 16–24 months of age
Tetanus toxoid	Two doses at 10 years and 16 years of age

**Table 2:** Kashmir mumps outbreak data.

Outbreak Number	Date of Occurrence	District	Village Affected	Number of Cases
1	17 July 2017	Pulwana	Lasidaban	10
2	24 July 2017	Shopian	Naserpora	25
3	27 July 2017	Shopian	Tukroo	11
4	15 August 2017	Shopian	Pinjoora	20
5	21 August 2017	Kupwara	Bowan Zachaldar	21
6	22 August 2017	Pulwama	Abhama	20
7	25 August 2017	Kulgam	Lirrow	7
8	26 August 2017	Pulwama	Dogripora	7
9	27 August 2017	Pulwama	Wahipora	8
10	28 August 2017	Shopian	Harman Nildoora	8
11	28 August 2017	Srinagar	Govt Middle School, Khawja Bagh Maloora	50
12	7 August 2017	Baramullah	Sheeri Najibhat	12
13	15 September 2017	Shopian	Batpora	10
14	16 September 2017	Kulgam	Yaripora	32
15	16 September 2017	Badgam	Babapora	19
